# Visual crowding illustrates the inadequacy of local vs. global and feedforward vs. feedback distinctions in modeling visual perception

**DOI:** 10.3389/fpsyg.2014.01193

**Published:** 2014-10-21

**Authors:** Aaron M. Clarke, Michael H. Herzog, Gregory Francis

**Affiliations:** ^1^Laboratory of Psychophysics, Brain, Mind Institute, Science Vie, École Polytechnique Fédérale de LausanneLausanne, Switzerland; ^2^Department of Psychological Sciences, Purdue UniversityWest Lafayette, IN, USA

**Keywords:** feed-forward, hierarchical models, feedback, object recognition, scene processing

## Abstract

Experimentalists tend to classify models of visual perception as being either local or global, and involving either feedforward or feedback processing. We argue that these distinctions are not as helpful as they might appear, and we illustrate these issues by analyzing models of visual crowding as an example. Recent studies have argued that crowding cannot be explained by purely local processing, but that instead, global factors such as perceptual grouping are crucial. Theories of perceptual grouping, in turn, often invoke feedback connections as a way to account for their global properties. We examined three types of crowding models that are representative of global processing models, and two of which employ feedback processing: a model based on Fourier filtering, a feedback neural network, and a specific feedback neural architecture that explicitly models perceptual grouping. Simulations demonstrate that crucial empirical findings are not accounted for by any of the models. We conclude that empirical investigations that reject a local or feedforward architecture offer almost no constraints for model construction, as there are an uncountable number of global and feedback systems. We propose that the identification of a system as being local or global and feedforward or feedback is less important than the identification of a system's computational details. Only the latter information can provide constraints on model development and promote quantitative explanations of complex phenomena.

## 1. Introduction

A common approach to understanding vision is to identify whether a particular aspect of visual perception involves “local” or “global” processing. Local processing suggests that the information needed for some behavioral task is determined predominately by information that is spatially close to the target stimulus. Global processing suggests that information processing is influenced by elements that may be distant from the target. Distinguishing between visual processing as being local or global has long been an important aspect of the Gestalt approach to perception (see the review by Wagemans et al., 2012). The local vs. global distinction also plays an important role in characterizing the flow of information in visual cortex (e.g., Altmann et al., 2003) and identifying the order of processing for natural scenes (e.g., Rasche and Koch, 2002; Cesarei and Loftus, 2011).

Likewise, many investigations try to identify whether visual processing involves “feedforward” or “feedback” processing. In a feedforward system the information flows in one direction, while in a feedback system the information flowing back and forth within and between areas can alter the processing at a given cortical location. In neuroanatomical studies, feedback processing is sometimes referred to as recurrent processing or re-entrant processing (especially when it involves information from higher cortical areas projecting to lower visual areas). Since feedforward processing tends to be easier to model, interpret, and compute than feedback processing, it is often the starting point for computational and neurophysiological theories and serves as a standard comparison for subsequent studies that explore feedback effects. For example, Hubel and Wiesel (1962) proposed a local feedforward model that accounted for the properties of simple and complex cell receptive fields, and subsequent studies then proposed the existence of non-classical receptive fields by demonstrating effects of feedback or global processing (e.g., Von der Heydt et al., 1984; DeAngelis et al., 1994; Freeman et al., 2001; Harrison et al., 2007). Likewise, a popular theory of visual processing proposed that both a rapid feedforward sweep and a slower recurrent process is involved in different behavioral tasks to different degrees (Lamme and Roelfsema, 2000; Lamme, 2006), and many studies have explored whether particular phenomena depend on one or the other processing approach. Examples include Altmann et al. (2003) reporting evidence for feedback processing in an fMRI study of perceptual organization; Enns and Di Lollo (2000) arguing that some forms of visual masking require re-entrant signals that represent objects; Juan and Walsh (2003) using TMS to argue that the representation of information in area V1 is influenced by feedback from other areas; and Keil et al. (2009) reaching a similar conclusion for emotionally arousing stimuli using an ERP study.

**Figure 1:**
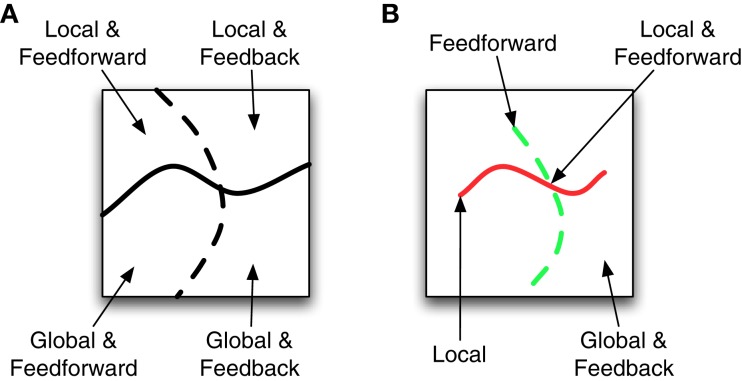
**Two possible spaces of models that vary as local or global and feedforward or feedback. (A)** Different model types are divided into roughly equal sized regions. **(B)** Models with local or feedforward attributes correspond to lines in the space. All remaining models use global and feedback processing.

Experimental vision science is full of many other examples of investigations into local vs. global and feedforward vs. feedback processing, and we generally agree with their methods and conclusions. However, we are less convinced that these characterizations are especially useful for developing models of visual perception that might account for observed behavioral phenomena, and we suspect that the benefits of the local vs. global and feedforward vs. feedback dichotomies have been somewhat overstated. The seeming appeal of investigations that distinguish between local vs. global and feedforward vs. feedback processing may derive from a misunderstanding about the general properties of complex systems. Figure 1A schematizes one way of conceptualizing model space. The solid wavy line separates local models from global models while the dashed line separates feedforward models from feedback models. Under such a model space, identifying whether a system requires local or global processing divides the possible number of models nearly in half. Likewise identifying whether a system requires feedforward or feedback processing again divides the number of possible models in half. If the model space were as dichotomous as in Figure 1A, then investigations about the local vs. global or feedforward vs. feedback nature of visual processing would be very beneficial to modelers.

However, the characterization in Figure 1A cannot be correct because there must necessarily be fewer feedforward and local systems than feedback or global systems (e.g., every feedforward system can be augmented with multiple types of feedback), so the model space depicted in Figure 1B is closer to reality. Here the local models are characterized by a thin red line and the feedforward models are characterized by a thin dashed green line. The class of local and feedforward models is the small intersection of these lines, while global and feedback models correspond to almost everything else. If this perspective of the model space is correct, then scientists gain a lot of information by knowing a system uses local (Weisstein, 1968) or feedforward processing (VanRullen et al., 2001), but they gain very little information by knowing the model uses global and feedback processing.

Our argument is not that distinctions between local and global or feedforward and feedback processing provide *no* information about the properties of the visual system; but if Figure 1B is correct, then such distinctions will not generally provide sufficient constraints to promote model development for the identified effects. While this limitation may already be clear to many modelers, it seems that some experimentalists do not fully understand that such distinctions provide very little guidance for model development. Part of the problem is the underlying textbook assumption that there is *one* standard feedforward model and another standard feedback model, which implies that all we have to do is perform an experiment to see which type of model better describes task performance. It is indeed true that there are successful and popular feedforward and feedback models. The feedforward model of Riesenhuber and Poggio (1999), for example, has been used successfully for things like fast-feedforward object recognition or scene classification (e.g., Hung et al., 2005; Serre et al., 2005, 2007a,b; Poggio et al., 2013). Similarly, the feedback model of Grossberg (e.g., Grossberg and Mingolla, 1985) has spawned a multitude of subsequent publications (e.g., Grossberg and Todorovic, 1988; Grossberg and Rudd, 1989; Grossberg, 1990; Francis et al., 1994; Francis and Grossberg, 1995; Dresp and Grossberg, 1997; Grossberg, 2003; Grossberg and Howe, 2003; Grossberg and Yazdanbakhsh, 2003; Grossberg et al., 2011; Foley et al., 2012). Clearly there is an important role for both types of model architectures. However, the success of these models is not simply because of their feedforward or feedback architecture. Even these “popular” models involve parameter variations and additional stages from one paper to the next that make them suitable for modeling one experimental data set, but not another. Moreover, there exists a broad continuum of models that are designed to model various phenomena and include various amounts of feedforward and feedback processing, or local and global processing, and that are all different. In this sense, there is not really a “standard” model for the visual system. Even V1 receptive field models are vast and varied, including such models as Gabors (Gabor, 1946; Jones and Palmer, 1987), balanced Gabors (Cope et al., 2008, 2009), difference of Gaussians (Sceniak et al., 1999), oriented difference of Gaussians (Blakeslee and McCourt, 2004; Blakeslee et al., 2005), the log-Gaussian in the Fourier domain (Field, 1987), and many more, all of which produce similar, but distinctly different effects when applied to natural images and lab illusions. Moreover, V1 receptive fields comprise just the first step in a model of visual cortex. Thus, no “standard” models exists for either feedforward or for feedback architectures, and similarly for a local or a more global connection architecture. Simply specifying one or the other type of architecture is not helpful for many modeling projects. To demonstrate our point, we consider empirical data from studies of visual crowding that show a clear non-local effect, and that likely require feedback mechanisms to enable perceptual grouping. We then describe the properties of three plausible models: one that can be considered to be feedforward and global, one that can be considered to be feedback and global, and one that can be considered to be feedback and global with a clear interpretation of perceptual grouping. We show through computer simulations that none of these models can account for the empirical findings that motivated them. This result suggests that we need to stop focusing on unhelpful dichotomies such as local vs. global and feedforward vs. feedback and instead should explore other properties of visual perception that help identify robust computational principles.

## 2. Visual crowding as an example

In visual crowding the discrimination of a target stimulus is impaired by the presence of neighboring elements. Crowding is ubiquitous in human environments. Even while you read these words, the letters appearing in the periphery of your visual field are crowded and largely unintelligible. Crowding can even be life-threatening in driving situations where a pedestrian can become unidentifiable by standing amongst other elements in the visual scene (Whitney and Levi, 2011). Moreover, visual crowding has been used to investigate many other aspects of perceptual and cognitive processing including visual acuity (Atkinson et al., 1988), neural competition (Keysers and Perrett, 2002), and awareness (Wallis and Bex, 2011).

**Figure 2:**
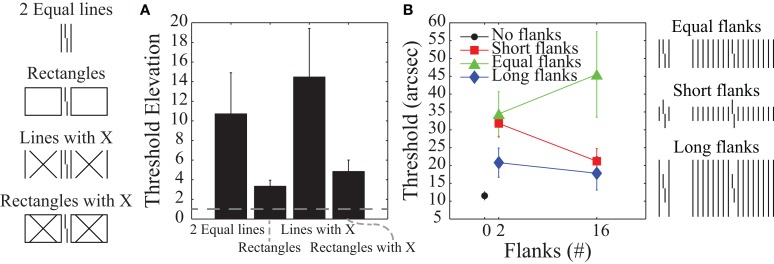
**Human data where higher values indicate stronger crowding. (A)** Threshold elevations for the stimuli shown on the left. Fixation was 3.88° to the left of the Vernier target, which was 84 arc min tall. Even though all conditions include vertical flanking lines on either side of the target vernier, there are dramatic differences in crowding. Such findings indicate global rather than local effects for crowding mechanisms. **(B)** Thresholds for the stimuli shown on the right. Here fixation was centered on the Vernier target. Varying the length and number of flanking lines shows that crowding increases when the target vernier groups with the flankers (as in the equal length condition). Such grouping effects indicate feedback processing. The plots are based on data from Manassi et al. (2012) and Malania et al. (2007).

The most popular models of crowding are local and feedforward models in which deteriorated target processing is due to information about the target being pooled with information about the flankers (e.g., Parkes et al., 2001). Although such pooling mechanisms are the default interpretation of crowding effects, recent studies have suggested that crowding involves global (rather than local) and feedback (rather than feedforward) processing (Malania et al., 2007; Levi and Carney, 2009; Sayim et al., 2010; Livne and Sagi, 2011; Manassi et al., 2012, 2013). Figure 2 schematizes eleven different types of stimuli where the task is always to identify the offset direction of a central target vernier. Figure 2A shows human vernier offset discrimination threshold elevations (relative to a no-flanker case), where larger threshold elevations indicate more crowding (from Manassi et al., 2012). The stimuli used are depicted on the far left-hand side of the figure. In all cases, the vernier is flanked by two vertical lines whose length matches the vertical extent of the vernier. The data in Figure 2A indicate that the different flanker types do not produce equivalent crowding despite the identical neighboring lines. Although the flanking lines alone or with an “X” produce substantial crowding, there is very little crowding when the very same lines are part of a larger structure. A “local” mechanism, such as pooling, would predict similar (or stronger) crowding with the additional contours in the rectangle configurations. The observed decrease in crowding suggests that the phenomenon cannot be explained by local interactions between stimuli.

Figure 2B shows human vernier thresholds (Malania et al., 2007) that have also been used to argue for feedback processing. Different experimental conditions varied the lengths of the flanking lines (shorter than, equal to, or longer than the vernier) and the number of flanking lines (0, 2, or 16). For the equal-length flankers, an increase in the number of flankers leads to stronger crowding, while for the short- and long-flanker lines, an increase in the number of flankers either reduced crowding or produced essentially no change. The argument for feedback processing has two parts. First, the data for the different conditions in Figure 2B suggest that crowding is strongest when the target vernier perceptually groups with the flankers (e.g., 16 equal-length flankers) and it is weakest when the target is perceptually segmented from the flankers (e.g., 16 short or long flankers). A sense of these grouping effects can be gained by looking at the schematized stimuli at the far right of Figure 2B. Second, perceptual grouping seems to require systems with feedback processing (e.g., Grossberg and Mingolla, 1985; Herzog et al., 2003; Craft et al., 2007; Hermens et al., 2008; Francis, 2009; Kogo et al., 2010). In particular, as Manassi et al. (2013) noted, the properties of crowding seem to defy low-level feedforward models based on stimulus energy or similar concepts (although they did not attempt to model their results). In their experiments they had subjects perform Vernier offset discrimination tasks and showed that when holding local information constant, global stimulus information still influenced thresholds. Thus, local information must have been propagated globally. Further experiments showed that this local-to-global information propagation takes time, implying feedback and recurrent processing.

Since the crowding data in Figure 2 indicate a role for global rather than local and feedback rather than feedforward processing, we wanted to use this knowledge to help develop a model of visual processing that accounted for crowding. Several models for crowding exist in the literature (e.g., Wilkinson et al., 1997; Balas et al., 2009; Greenwood et al., 2009; Van den Berg et al., 2010; Freeman and Simoncelli, 2011). Our intent here is not to classify these models as feedforward/feedback or local/global, and see how well they work, but rather to examine some clear examples of models using various amounts of feedforward/feedback and local/global processing and demonstrate the utility (or lack thereof) of knowing that a phenomena requires feedforward/feedback or local/global processing for modeling behavioral results. As the following sections demonstrate, we found this knowledge to be inadequate, and we believe that the modeling challenges here reflect issues that also apply to other phenomena and modeling efforts. Although it is possible that we happened to simulate models that are poor fits for the phenomena, we deliberately investigated models that have successfully modeled similar stimuli and phenomena, so we believed that they might also be able to account for the empirically observed crowding effects.

## 3. A feedforward global model: Fourier analysis

Researchers have suggested that it is useful to describe visual processing in terms of Fourier components (Campbell and Robson, 1968; De Valois et al., 1982). Luminance values at different (*x*, *y*) coordinates in the pixel plane can be converted to weights for different sine wave frequencies' amplitudes and phases. In principle, such a transformation does not lose any information, so if the luminance image contains information about the offset direction of a vernier, then so does the Fourier representation. However, such information can become degraded or lost when important frequencies or phases are filtered out of the representation. Such filtering can be justified on neurophysiological grounds (Campbell and Robson, 1968; De Valois et al., 1982) or be chosen to explain perceptual phenomena. For example, multi-scale filtering can explain a variety of brightness illusions (Blakeslee and McCourt, 1999, 2001, 2004; Blakeslee et al., 2005).

Fourier decomposition can be considered to be a feedforward process, with a bank of filters that are tuned to different frequencies, orientations, and phases (Fourier, 1822), and such an interpretation is a common first-approximation to cortical visual processing (Campbell and Robson, 1968). On the other hand, Fourier analysis is decidedly global rather than local in the sense that the weights assigned to different frequencies are based on the pattern of luminance values across the entire image plane (Rasche and Koch, 2002; Cesarei and Loftus, 2011). It is also global in the sense that a filter that suppresses some frequencies will influence representations of luminance values across the entire image plane when the frequency weights are converted back to an image representation.

**Figure 3:**
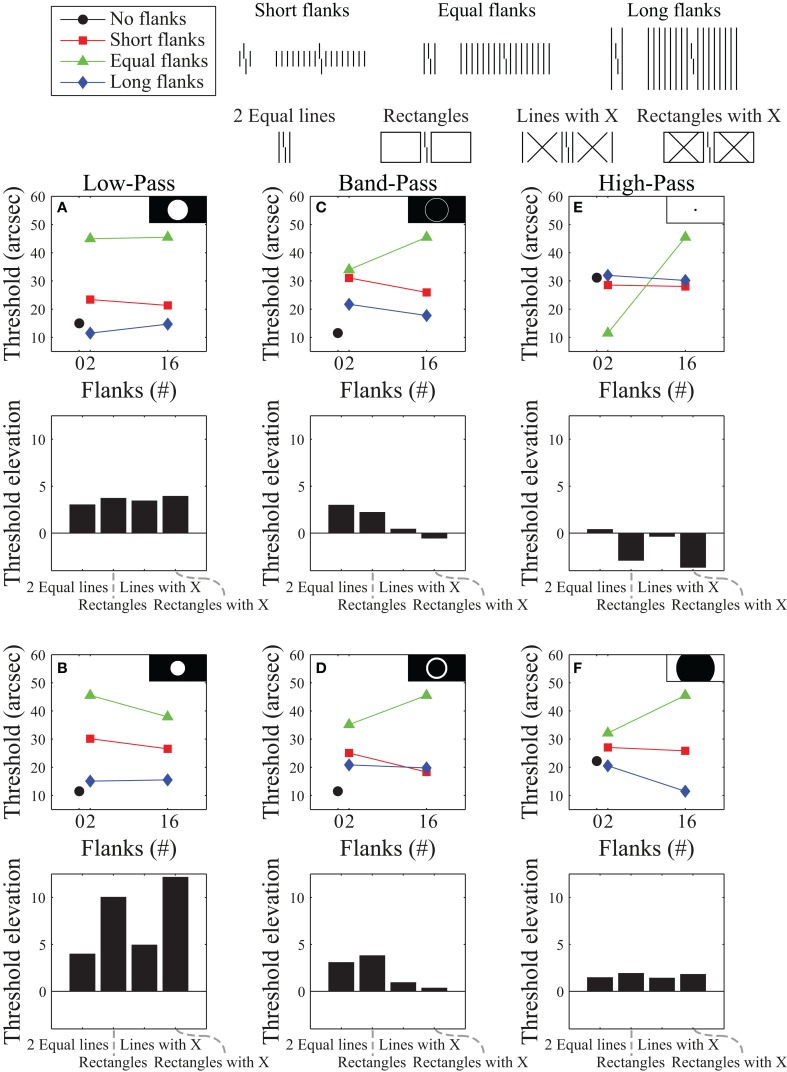
**Simulation results using a Fourier model for the stimuli that produced the data presented in Figure 2**. Model results are plotted for representative low-pass filters **(A,B)**, band-pass filters **(C,D)**, and high-pass filters **(E,F)**. Black and white insets show which frequencies were passed (white areas) and which frequencies were suppressed (black areas) in Fourier space (with lower frequencies in the center and higher frequencies near the edges). The top row of subplots shows the best performance obtainable (using brute-force exhaustive search for the smallest sum of squared residuals against the human data) with each filter type. The bottom row shows results for filtering functions selected from different parts of the space - illustrating the variability of results obtainable with each filter type. The best *overall* performance we could obtain with this model is shown in **(C)**.

We developed a model that applies a Fourier analysis to the image, filters out a subset of spatial frequencies, applies a Fourier synthesis to construct a filtered version of the image, and then compares the output with a template for discriminating right- from left-offset verniers. The difference between template matching results for the left- and right-offset verniers are subtracted and the difference is then inverted and linearly scaled to the range of the human data. Model details are provided in the Supplementary Material. To try to match the empirical data, we examined various filtering schemes, including high-pass filtering, band-pass filtering, and low-pass filtering. Figure 3 shows a representative selection of results for the stimuli used to produce the data in Figure 2. Even though they all allow for global processing, many of these frequency filtering functions produce results that differ dramatically from the human data. Within each filtering scheme we identified the filter parameters that yielded the smallest sum of squared residuals between the model and human data from Figure 2 by exhaustive, brute-force search over the entire parameter space. Figure 3C, shows the best fit overall, which was obtained with a band-pass filter.

This best filter mask for the data used in Figure 3C does a reasonably good job at reproducing the human data from Figure 2B, but it does a poor job reproducing the human data shown in Figure 2A. Although the model roughly follows the pattern of the data for the two-line flanker and rectangle conditions, it predicts very little threshold elevation (and even threshold improvement) for the conditions with an “X” superimposed over the flanker regions. These predictions do not match the empirical data. The other filter functions also fail to reproduce the human data for these flanker conditions.

**Figure 4:**
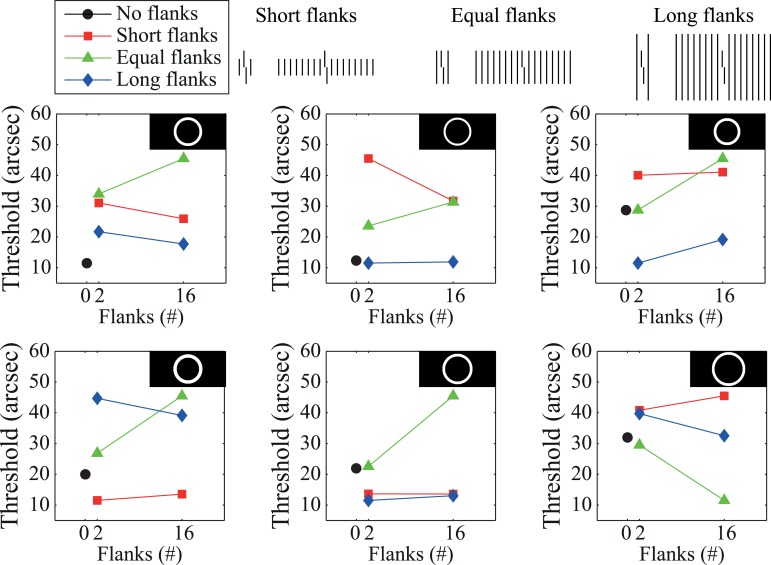
**Predicted behavior of the Fourier model for filters that slightly differ from the optimal band pass filter (shown in the upper left graph)**. Small changes in the band pass filter's center frequency and/or bandwidth lead to dramatic changes in the model's behavior.

Moreover, the best filter is fragile in that small changes in bandwidth and/or center frequency lead to very different model predictions. This fragility is demonstrated in Figure 4, which shows model performance for band pass filters that are only slightly different from the filter that produces the best fit to the empirical data in Figure 2B. This behavior is surprising since Fourier models generally tend to fail gracefully with small deviations from the optimal filter parameters. The wildly varying model behavior suggests that the good fit exhibited in Figure 3C reflects over-fitting rather than a mechanistic explanation of the behavior. Overall, the model fails to account for the human data in a robust way. Such a failure occurs even though the model is inherently global in terms of processing, and thus satisfies one of the requirements seemingly needed to account for crowding effects. We cannot definitively claim that all Fourier-type models cannot account for crowding effects, but it seems that a good model does not easily appear simply because it has global processing.

## 4. A feedback model: Wilson-Cowan neural network

We next considered a model that derives its key properties from the recurrent nature of information processing in a cooperative-competitive neural network. Variations of this kind of model have successfully accounted for visual masking data (Hermens et al., 2008) using stimuli very similar to those in Figure 2. The model first convolves the input image with an on-center, off-surround receptive field mimicking processing by the LGN. Next, the input activations are fed into both an excitatory and an inhibitory layer of neurons. Each layer convolves the input activations with a Gaussian blurring function and propagates activity over space with increasing time. The layers are reciprocally connected such that the excitatory units excite the inhibitory units and the inhibitory units inhibit the excitatory units. Details of the model, its filters, and its parameters can be found in Hermens et al. (2008) and Panis and Hermens (2014). Although the filters are local, the strength of activity at any given pixel location partly depends on the global pattern of activity across the network because of the feedback connections. When played out over time in a backward masking situation with stimuli similar to those in Figure 2, Hermens et al. (2008) showed that masking strength decreased as the number of flanking elements increased. More generally, the feedback in the network functions somewhat like a discontinuity detector by enhancing discontinuities and suppressing regularities. Panis and Hermens (2014) showed similar behavior for stimuli that produce crowding.

**Figure 5:**
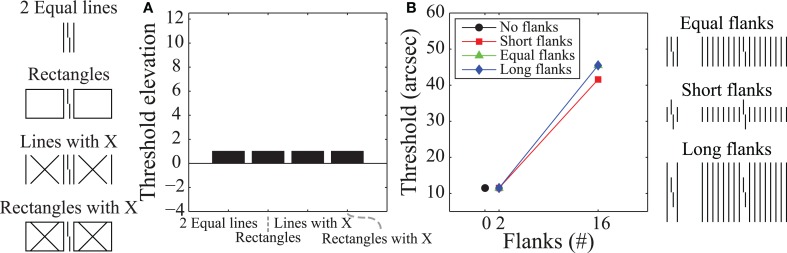
**Results obtained using the model of Hermens et al. (2008) on the stimuli shown in Figure 2**. Compared with the human data plotted in Figure 2 this model does a poor job at capturing human performance, despite using feedback signals that propagate information globally. Parts **A** and **B** of this figure correspond to parts **A** and **B** of Figure 2.

Since the model includes lateral feedback that promotes global processing, it satisfies the requirements identified above as “necessary” to explain crowding's effects. Moreover, the models parameters were previously optimized for one stimulus, and then the model was validated by applying it to novel stimuli without further parameter optimization (Hermens et al., 2008). Thus, we would expect that any additional stimulus conditions that we apply this model to should require no further parameter optimization. We analyzed the model's behavior in response to the stimuli used to generate the findings in Figure 2 but found that the model performs poorly overall (Figure 5). In particular, the model produces virtually no difference between any of the conditions shown in Figure 5A. Figure 5B shows that the model also fails to reproduce the human data plotted in Figure 2B. Here, the model produces no substantial differences between the different flanker length conditions, it produces no crowding for the case where there are two flanking lines (thresholds are the same as in the un-flanked case), and model thresholds always go up as an increasing function of the number of flankers (contrary to the human data).

Even though the model has previously accounted for perceptual effects with similar kinds of stimuli and has strong feedback and global effects, the model simulations reported here do not account for the crowding effects in Figure 2. We cannot claim that the model architecture is fully rejected, as different filters and parameters may produce different model behaviors. Nevertheless, it is clear that global and feedback processing by themselves do not sufficiently constrain model properties relative to the observed crowding effects.

## 5. A feedback model with perceptual grouping: LAMINART neural network

The previous simulations indicate that a model needs additional constraints beyond just feedback and global processing. We next consider a model that has many additional constraints, the LAMINART model that has been proposed by Grossberg and colleagues (Raizada and Grossberg, 2001). The model is very complex and involves neural signals that interact across retinotopic coordinates, across laminar layers within a cortical area, and across cortical areas V1, V2, and V4. Various forms of the model account for neurophysiological and behavioral data related to depth perception (Grossberg, 1990; Grossberg and Howe, 2003), brightness perception (Grossberg and Todorovic, 1988), illusory contours (Grossberg and Mingolla, 1985), backward masking (Francis, 1997), and many other effects (Grossberg, 2003; Grossberg and Yazdanbakhsh, 2003). In particular, model simulations in Francis (2009) used stimuli very similar to those in Figure 2 to successfully account for a variety of backward masking effects. An integral part of the model explanations involved a form of perceptual grouping, which was indicated by the presence of illusory contours connecting elements within a group. Consistent with the ideas derived from Figure 2, these model grouping processes use feedback to generate global effects.

**Figure 6:**
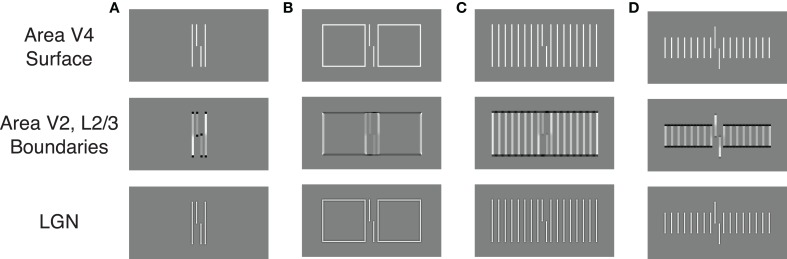
**Simulation results for the LAMINART model of visual perception**. The bottom row indicates the activity of LGN cells and largely reflects the stimulus. The middle row schematizes the activity of orientation-sensitive neurons (dark gray to black indicates activity of a horizontally-tuned cell, light gray to white indicates activity of a vertically tuned cell). The top row schematizes the activity of neurons that represent surfaces with perceived brightness. Judgments of target offset are based on the top row activities. **(A–D)** demonstrate the model's behavior for several different types of flankers.

**Figure 7:**
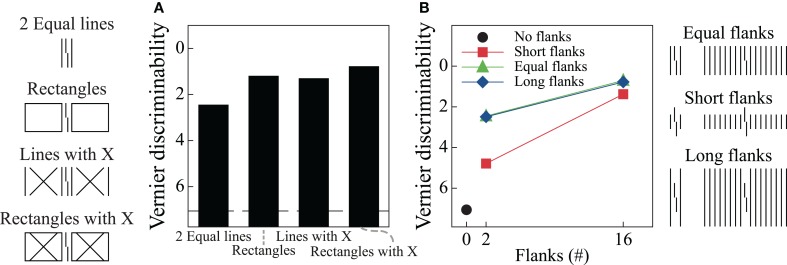
**Simulation results for the LAMINART model of visual perception**. The dashed line indicates discrimination for the target vernier by itself. Vernier discrimination is plotted in reverse for easy comparison with the threshold measures reported in Figure 2. Overall, the model behavior does not agree with the empirical data. Parts **A** and **B** of this figure correspond to parts **A** and **B** of Figure 2.

The model proposes separate processing streams for boundary and surface information. Grouping effects mostly occur in the boundary system through formation of illusory contours that connect nearly collinearly oriented edges, and Figures 6A,B show simulation results for two of the stimulus conditions in Figure 2A. When the flankers are two lines, the model generates boundary signals that represent each stimulus line (Area V2, Layer 2/3) and these boundary signals constrain brightness signals that pass from the LGN to Area V4. As a result, the Area V4 representation is essentially veridical relative to the original stimulus. As described in the Supplementary Material, the model signals are connected to human performance with a template matching process that tries to distinguish between verniers shifted to the left or right. Crowding effects occur because the vernier template (whose width is five times the spacing between stimulus elements) integrates information from both the flankers and the vernier target, thereby reducing the signal-to-noise ratio for vernier discrimination. In this way, the model matches the empirical finding that two flanking lines can produce crowding. Figure 7A shows vernier discriminability (plotted in reverse for comparison with the threshold data) for this simulation, and it indicates that it is harder to identify a vernier with two flankers than to identify a vernier by itself (the dashed line).

Figure 6B shows the model's behavior when the flanking elements are rectangles. Although the local information is similar to that in the case of two flanking lines, the Area V2, Layer 2/3 cells respond quite differently by producing illusory contours that connect the two rectangles and the target vernier. Nevertheless, at the V4 filling-in stage, the perceptual representation is nearly veridical, and crowding occurs because the flanking elements again interfere with the vernier template matching calculations. Although the model has perceptual grouping, it incorrectly produces strong crowding where the empirical data indicate only weak crowding effects. These effects are indicated in Figure 7A, where the rectangle flankers condition indicates worse vernier discrimination than does the two equal-length flankers condition. Using flankers with an “X” produces the same pattern as for the conditions without an “X,” and the rectangles provide the strongest masking. The data in Figure 2A shows the opposite pattern for the rectangles.

Similar properties exist for the stimuli producing the findings in Figure 2B. Figures 6C,D show the model's behavior in response to sixteen equal and short flankers. Consistent with the arguments about grouping described above, in the equal-length case the model generates illusory contours that connect the flankers with the target, thereby collectively grouping the flankers and target together. At the filling-in stage, all of the elements are represented and there is strong crowding. Also consistent with the above arguments, grouping is different for the short flankers (similar behavior would occur for the long flankers), such that the flanking elements are connected by illusory contours but the target remains separate. However, such grouping does not lead to a release from crowding in the model. At the Area V4 filling-in stage the flanking elements still interfere with the vernier discrimination process, even though the boundaries indicate that the flankers and target are part of different perceptual groups. Figure 7B shows that the LAMINART model does not do a good job of matching the behavioral data in Figure 2B. This failure occurs even though the model includes feedback, has global effects, and contains grouping mechanisms that seem to operate much as recommended. Our claim is not that the model can be fully rejected by this failure, but we want to emphasize that a model with feedback, global processing, and mechanisms for perceptual grouping is not necessarily able to account for the observed human data.

## 6. What constraints does a model need?

The model simulations of crowding demonstrate that identification of global vs. local and feedback vs. feedforward processing does not necessarily promote the development of models that can account for human performance. We suspect the same kind of conclusion applies to models for many other visual phenomena. Although quantitative models of visual perception that account for visual processing often do include feedback and global processing (e.g., Bridgeman, 1971; Grossberg and Mingolla, 1985; Francis, 1997; Roelfsema, 2006; Craft et al., 2007; Kogo et al., 2010), this inclusion is often because such mechanisms provide specific computational properties that are needed to produce a functional visual system. The failure of the models discussed here relative to their success for other phenomena (e.g., backward masking) encourages a consideration of what kinds of constraints are useful for model development. It is unlikely that there is one single answer to this question, but we are willing to propose some ideas.

### 6.1. Global vs. local is about information representation

All models of visual processing involve encoding and representing information about the stimulus, and such a representation changes at various model stages so that some information is explicitly represented, other information is only implicitly represented, and some information is absent. A local model is one where the encoding of information about a certain position in visual space is modified only by information at nearby positions in space. In the case of crowding, the argument against local processing is that explicit or implicit information about the target vernier appears to be affected by stimulus characteristics that are spatially far away in an unexpected way (e.g., two flanking squares produce less crowding than two flanking lines).

Even when the argument for non-local effects is convincing, it does not specify exactly how information about the target should be represented in a global-effects model. The crowding models described here include different types of information representation and different types of global effects. The Fourier model transforms spatial information into spectra and then applies a filtering step that loses some information about the target (as well as information about the flankers). The Wilson-Cowan model represents visual information in spatial (retinotopic) coordinates and introduces global effects via recurrent lateral inhibition. The LAMINART model also represents information in spatial coordinates, and it generates global effects via long-range illusory contours that connect spatially disparate boundaries, which can alter the boundary representations of the target. In practice, none of these global mechanisms produce crowding effects that emulate the behavioral data, at least in the instantiations considered here.

It seems to us that the global vs. local processing issue is something of a “red herring” that ignores deeper questions about the representation of visual information. A model must encode visual information in a way that allows for local or global processing, and identification of this encoding and its representation is the real model challenge. For example, in the LAMINART model, the information at the V4 surface stage provides a representation of information that (for the stimuli considered here) is essentially the same as the stimulus. Although there are groupings among boundaries, they do not modify the representation of visual information that is involved in the vernier offset judgment. What appears to be needed is for the boundary groupings to segment the visual information so that the target is represented separately from the flankers. In this way, the target's offset could be discriminated with less interference from background elements. Francis (2009) described how such segmentation can occur for some visual masking situations that encode information about the target at the V4 surface representations in a different depth plane than information about the flankers. Such segmentation promotes good discrimination of the vernier offset. Foley et al. (2012) demonstrated that attentional effects could also produce similar segmentations in crowding conditions.

### 6.2. Feedforward vs. feedback is about model function

Many of the discussions about feedforward vs. feedback processing seem predicated on the notion that if information is available at a model stage, then it can be used for a relevant task. For example, if binocular disparity information is available at V1, then it can be used for making depth discriminations at this stage. However, this attitude does not consider the many ways that feedback processing can influence information processing. In general, feedback processing tends to produce one of five robust model functions.

1. Completion: Excitatory feedback can “fill-in” missing information and thereby make explicit information that is implicitly represented by other aspects of an input pattern. One example of such completion is the generation of illusory contours in the LAMINART model, where the model explicitly represents “missing” contours that are justified by the co-occurrence of appropriate contours that are physically present. Another example of such completion is in the convergence of a Hopfield (1982) network to states with active neurons that were not directly excited by the input but are justified by their association with other active neurons.
2. Competition: A combination of excitatory and inhibitory feedback can enhance differences in neural activity and, in extreme forms, generate winner-take-all behavior in a network of neurons (Grossberg, 1973). Such networks can suppress noisy or irrelevant information and enhance the representation of other information in the system. For example, Wilson et al. (1992) proposed a competitive neural network to explain vector summation in motion perception, where units tuned to a particular motion direction inhibit units tuned to the orthogonal motion direction.
3. Preservation: Excitatory feedback can allow signals to persist well beyond the physical offset of a stimulus (e.g., Öğmen, 1993; Francis et al., 1994). Inhibitory feedback can also play a role in preservation of information by suppressing incoming signals that might alter the current pattern (Francis, 1997, 2000). A combination of excitatory and inhibitory linear feedback can also preserve pattern representations even with large changes in overall intensity (Grossberg, 1973). Grossberg et al. (2011), for example, used preservation to extend flickering stimuli long enough through time to allow their temporally integrated signals to generate smooth motion percepts.
4. Uniformization: Some types of non-linear feedback can diminish differences in neural activity and lead to uniform activity (Grossberg, 1973). Such information-losing feedback is not commonly used in neural models.
5. Comparison: Appropriately structured excitatory and inhibitory feedback can generate a signal that indicates the degree to which two neural activity patterns differ. Such signals are helpful for larger architectures that need to detect changes or stabilize learning (e.g., Grossberg, 1980; Sutton and Barto, 1998; Di Lollo et al., 2000).

These different functions often require rather different feedback mechanisms that involve the distribution of excitatory and inhibitory relations, the relative strength of feedback and feedforward signals, and the form of signal transformation between neurons. Thus, model development requires a characterization of function in order to be able to properly implement feedback. Characterizing model function is, of course, very challenging and generally requires some kind of over-arching theoretical framework to guide the computational goals of the model. For example, a model of crowding that theorizes a role for perceptual grouping needs to indicate how elements in a scene are identified as being “grouped,” explain the mechanisms by which such distinctions are generated, and characterize how such representations influence target processing and decision making. A focus on such functional details may reveal that a certain form of feedback processing is critical for the model to reproduce the human behavior (Raizada and Grossberg, 2001), or it may reveal that the feedforward vs. feedback distinction is not as relevant as it first appeared (e.g., Francis and Hermens, 2002; Poder, 2013).

## 7. Conclusions

If the starting point of theorizing is that visual processing involves local interactions in a feedforward system, then it makes sense that investigations should explore whether such systems are sufficient to account for a given phenomenon. However, the modeling efforts presented here suggest that clear evidence of a role for global and feedback processing does not sufficiently constrain a model. At best, such investigations are only the starting point for model development, and further considerations are required concerning the details of information representation and model function. It might be easier to initiate theorizing by assuming global and feedback processing and then look for other more informative constraints such as task optimality, or perceptual completion.

## Author contributions

Aaron M. Clarke coded the simulations for the Fourier and Wilson-Cowan models. Gregory Francis coded the simulations for the LAMINART model. All authors contributed to the text.

### Conflict of interest statement

The authors declare that the research was conducted in the absence of any commercial or financial relationships that could be construed as a potential conflict of interest.
