# Supplementary material for: Visual crowding illustrates the inadequacy of local vs. global and feedforward vs. feedback distinctions in modeling visual perception
Source: Front Psychol. 2014 Oct 21;5:1193. doi: 10.3389/fpsyg.2014.01193 (PMC4204448; doi:10.3389/fpsyg.2014.01193)
Supplement: Supplementary file 1 [file DataSheet1.PDF]

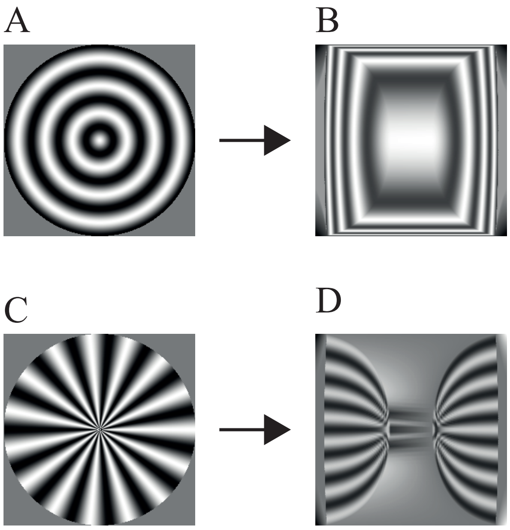

Figure 1. Illustration of the transformation taking retinal images (A & C) to their cortical representation (B & D) for concentric rings (A & B), and radial spokes (C & D).

APPENDIX

FOURIER MODEL

The Fourier model was implemented in MatLab® with a four-step procedure. In the first step, the stimulus was Fourier transformed, masked to filter out the unwanted frequencies (the masks were either low-pass, band-pass, or high-pass), and inverse Fourier transformed. In step two, the results from step one for each stimulus presented in Figure 2 were point-wise multiplied by an unmasked template made by subtracting an isolated left-offset vernier from an isolated right-offset vernier, yielding a "proportion rightward" model response for each stimulus. In step three, results for the stimuli containing a left-offset vernier were subtracted from the results for the same stimuli containing a right-offset vernier (small differences implying that the left- and right-offset verniers were indiscriminable in the given condition and large differences indicating that they were easily discriminable). Finally, in step four, the values produced by step three were inverted (to convert small stimulus differences into high thresholds) and scaled so that the minimum inverted difference corresponded to the minimum vernier offset threshold observed in Figure 2**B** and the maximum inverted difference corresponded to the maximum vernier offset threshold observed in Figure 2**B**. For simulating the results shown in Figure 2**A**, the results of step four were divisively normalized by the results of processing the vernier stimulus alone to arrive at threshold elevation. Matlab® source code for this simulation may be found at:
`https://osf.io/t25cr/?view_only=0a655dbd88a14c98a14380e0a293b528`.

8 FOURIER ANALYSIS WITH CORTICAL SCALING

The frequency content of cortical image representations is affected by cortical magnification and peripheral scaling. These factors tend to compress information in the retinal periphery (removing high-spatial frequency information), while magnifying foveal information (preserving high-spatial frequency information; see Figure 1 for examples). This is particularly relevant for vernier acuity tasks. Levi et al. (1985) for example, showed that vernier acuity with a crowded target declines with eccentricity, but is constant when cortical magnification is taken into account (Levi et al., 1985). Hence, these factors may be relevant for our Fourier model.

We thus re-ran our previous Fourier analysis model, but first applying cortical magnification and peripheral scaling to the retinal image, prior to all other steps in the model. Schwartz (1980) proposed a simple model that applies the necessary pre-processing transformations. Here, retinal image coordinates are transformed into cortical coordinates via the equation $w = a\log_e(z + b)$, where w is a complex number whose real and imaginary parts represent x- and y-coordinates in the image plane respectively, and $z = re^{i\theta}$, where r denotes retinal eccentricity and θ denotes angle from the meridian. The constants a and b have been determined empirically to be roughly 19.2 and 2 respectively (Schwartz, 1980). The results of applying these transformations prior to our Fourier model are plotted in Figure 2. Here it can again be seen that simply employing a feedforward, hierarchical model with the appropriate transformations to convert the retinal image into a cortical image is insufficient to reproduce the human data. This again suggests that the model lacks a mechanistic explanation of the observed human behaviour. Matlab® source code for this simulation may be found at:
`https://osf.io/t25cr/?view_only=0a655dbd88a14c98a14380e0a293b528`.

WILSON-COWAN MODEL

Details of the model equations and parameters can be found in (Hermens et al., 2008). The current simulations used the same model, with changes only to the stimuli and their durations. Specifically, the stimulus durations were increased to 300 ms (as in Malania et al., 2007) and model responses were read-out at the end of the stimulus duration. Matlab® source code for this simulation may be found at:
`https://osf.io/wqn5x/?view_only=69ba37a5463645f89fca15d569f8f977`.

LAMINART MODEL

Details of the model equations and parameters can be found in (Francis, 2009). The only changes to the simulations were to generate stimuli that matched those used to produce the data in Figure 2. Model performance was derived from templates designed to match a vernier shifted to the left, M_L, or right, M_R. For computing vernier discriminability, the templates were centered on the target vernier and uniformly summed signals within 30 $\times$ 40 pixels across a region covering the top left and another region covering the bottom right of the target. The width is five times the spacing between stimulus elements and the height is twice the length of the target vernier offset line. Such a large template allows for crowding when flanking elements overlap the template. A signal for the vernier direction being shifted to the right at time t was then computed as a contrast

$$C_R(t) = \frac{M_R(t) - M_L(t)}{0.01 + M_R(t) + M_L(t)}, \tag{1}$$

where the constant 0.01 avoids division by zero. Although the model in (Francis, 2009) includes two distinct depth planes, near and far, for the current simulations activity was only present in the far depth plane. The evidence for the vernier being shifted to the right was an integration of contrast energy across time

$$E_R = \int_{\tau_1}^{\tau_2} C_R(t)dt \tag{2}$$

where τ_1 indicates the start of the trial and τ_2 indicates the end of the simulation for a trial (when the target signals disappear). Larger values of E_R indicate better detection of the vernier direction and should correspond to smaller thresholds.

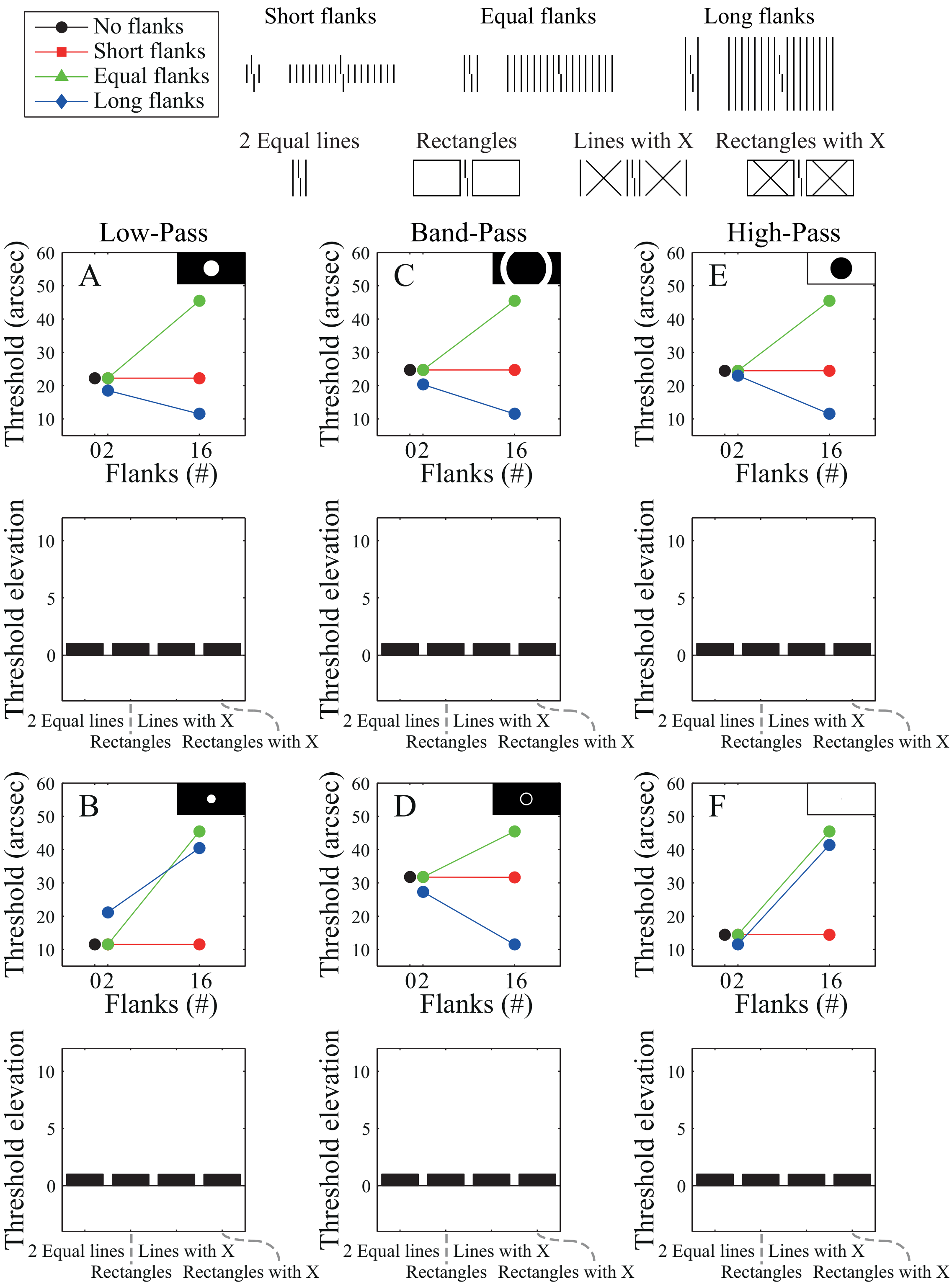

Figure 2. Repeated simulation using the Fourier model, but with cortical magnification and peripheral scaling taken into account prior to all other model steps. Plotting follows the same conventions as for Figure 3.
